# The “Standard of Medical Education”

**Published:** 1871-08

**Authors:** 


### Editorial Department.

### The "Standard of Medical Education."

The "Medical Education" hobby upon which a great many medical editors have written so freely, for years past, and upon which the time of Medical Associations and ambitious medical men has been so largely expended, is not yet devoid of practical living interest. We have never indulged our fancy in portraying the glorious altitude that the profession will hereafter occupy when its "Educational Standard is fully raised," but have engaged ourselves mainly in studying means for elevating it. This editorial will be very short, and is designed only to suggest what appears to us as the more practical method of elevating the profession. It seems that writers and orators have universally, and almost without exception, regarded the curriculum of the College course as the "Standard of Medical Education," which it was so desirable to have raised, and this is the view which we want to correct. Medical education, is commenced, it is true, at the College; here, is laid the foundation upon which can, in after life be raised a superstructure which is the true "standard." However perfectly this foundation is laid, if it is regarded as the standard of medical education, how fatal is the mistake. Physicians who think that the standard is raised at graduation, (and this seems the general impression) generally cease labor, and it actually becomes their standard. Whoever is quite familiar with the customs and habits of young graduates in medicine, has learned that it is not always the easiest to learn or the most ready to observe, or even those of highest mental culture and greatest attainment, who build the highest standards of medical knowledge; the after habits of life determine the question in much greater degree. Some one in the medical world has written learnedly upon this subject, and suggested that where a man was graduated might indicate how well he was educated, and that the initial letters of his *Alma mater* might properly be added to the general title.

The medical knowledge of a physician is but very partially obtained during his college course, so small a part of it is gained during the years of his pupilage that it may truthfully be said that he has only learned how to commence his search for medical knowledge. The foundation stones are laid, but the "elevation" remains for after life. It is idle to talk very much more upon the general proposition that to elevate the professional standard we must depend upon the medical schools of the country alone, to do the work. We are among the very last to oppose any efforts which the schools may make, or pretend to make, for more thorough training; the pretence, even, may be productive of some good, however destitute of reality it may prove; but we, at the same would remind the medical public, how little available knowledge of disease is really gained during pupilage, diverting if possible, attention from medical schools as alone the common source of all our faults.

Our primary and professional course of study is of the greatest importance, and cannot be too highly appreciated in making up the character and influencing professional standing and life. But if our schools required as preliminary, graduation in Literary and Scientific schools, and then extend their own terms to as many months and to as many years as they please, they could never graduate a creditable *alumnus*, if he was made to believe that his education was complete and that his *alma mater* had actually "raised his standard" of medical education. The medical men of our country, those who have taken places in actual service, are the ones who can raise our professional standard. Our colleges can do something, and I believe have done much, very much, in this great reform. To their organization and successful working are we indebted for rapid progress in our professional knowledge, but it is not for them alone to raise our standards. The most stupid graduates of our colleges have often gained attainments and position, so as to far outrival the more educated and thoroughly qualified. Three or four years only commences our education; its standard is not measured by it, its completion is not even faithfully foreshadowed by it. Let those who expect so much in one direction, turn their attention to other and more potential causes of our low standards. The idea that graduation means complete education, and that nothing remains but to use their already acquired knowledge when urgent necessity requires, is one of the main reasons why our professional status is so low; while this remains it will always be low.

The popular demand for high standard of medical knowledge is not sufficiently obvious. The public mind is either not capable of appreciating it, or has never been educated to rightly estimate its value, still, we believe, that finally physicians are estimated by the public at about what they are worth.

Who has ever written or said a word about elevating medical education but has commenced and ended with what educational institutions do, or do not do? This has been carried so far that physicians regard themselves as exempt from the necessity of personal effort, and wonder that the standard of medical education is so low—think it very strange that our medical colleges do not raise it up. It is right that the profession require somewhat of the medical colleges; it is equally just that the colleges require somewhat also of the profession; "a poor rule that don't work both ways." We shall always have in our profession men of all grades of attainments, the same that will prevail in other professions and callings. We shall continue to graduate as formerly, classes of students of varied capacities and attainments, some will continue to add to their acquirements year by year, during life, and thus in time will become educated physicians. Some will graduate and grow to know less and less if possible, and some will stand still; the most giganantic failure of all being observed in those who have graduated with credit, but with the impression that medical education is obtained in college, and are not made to understand that twenty years or more of constant faithful study, is indispensable to high standard of medical knowledge.

MORAL.—Every doctor must raise his own standard of education.

